# Long-term follow-up of whole lung lavage in patients with pulmonary alveolar proteinosis

**DOI:** 10.3892/etm.2014.1788

**Published:** 2014-06-18

**Authors:** XIAOYAN ZHOU, GUOCHU LU, ZHEN YU, FEI GAO, TAO BIAN

**Affiliations:** Department of Respiratory Medicine, Wuxi People’s Hospital Affiliated to Nanjing Medical University, Wuxi, Jiangsu 214023, P.R. China

**Keywords:** pulmonary alveolar proteinosis, whole lung lavage, follow-up

## Abstract

Pulmonary alveolar proteinosis (PAP) is a rare disorder characterized by intra-alveolar accumulation of lipid and proteinaceous material. While a small subset of patients with PAP spontaneously improve or even undergo disease remission, the majority of patients develop persistent or progressive disease. Numerous therapies have been used to treat PAP over the years; however, at present, whole lung lavage (WLL) remains the gold standard treatment for PAP. In the present study, data were accumulated from a cohort of patients with PAP (n=11) between 2003 and 2011 at the Wuxi People’s Hospital Affiliated to Nanjing Medical University. The disease affected males and females with a ratio of 2.7:1 and all the males were current or previous smokers. The disease severity score (DSS) of the patients was mainly distributed in DSS 4 or DSS 5. All the patients underwent WLL at least once, with one patient undergoing WLLs twice and another patient three times. The clinical features, arterial blood gas and pulmonary function of the patients, were assessed prior to and following the lavage. WLL resulted in a significant improvement in symptoms, radiographic features, PaO_2_, D(A-a)O_2_ and DLCO in patients with PAP, while pulmonary ventilation function did not significantly improve. WLL appears to be an effective approach for the treatment of PAP and leads to an improvement in survival rate.

## Introduction

Pulmonary alveolar proteinosis (PAP) is a rare diffuse pulmonary disease initially described by Rosen *et al* in 1958 (1). The clinical features of PAP include the accumulation of periodic acid-Schiff (PAS)-positive lipoproteinaceous material, predominantly phospholipid surfactants and surfactant apoproteins, in the distal air spaces, resulting in impaired gas transfer.

Patients with PAP may suffer from progressive dyspnea and cough, which may be accompanied by exacerbated hypoxia. Its course is variable, ranging from progressive deterioration to spontaneous improvement (2). Radiographic analysis of the disease revealed ground-glass opacities with multiple bilateral, irregular and dense inhomogeneous lesions, which have a ‘crazy-paving’ pattern on computed tomography (CT) scans (2). Confirmation of diagnosis of PAP is achieved through using typical electron-microscopic findings in sputum, lung washings and lung biopsy specimens, without which the condition is frequently mistaken for interstitial lung disease, particularly sarcoidosis.

For the treatment of PAP, various different therapies have been utilized over the years, for example, postural drainage, antibiotics and intermittent positive pressure breathing (3). Major advances were achieved in the late 90s by Kitamura *et al* (4) and Tanaka *et al* (5), who identified the presence of autoantibodies neutralizing granulocyte-macrophage colony-stimulating factor (GM-CSF) in the serum and lung tissue of patients with idiopathic PAP, and since then various specific treatments have been attempted or proposed (6). However, despite this, the standard treatment remains whole lung lavage (WLL), which was modified following the original description by Ramirez *et al* (7) in 1963.

In the present study, 11 patients with PAP who were treated with WLL were followed up, and their clinical manifestations, treatment outcomes and prognosis were reviewed.

## Patients and methods

### Patients and study design

The present study was conducted at the Wuxi People’s Hospital Affiliated to Nanjing Medical University (Wuxi, Jiangsu, China) for the diagnosis and therapy of PAP in China. Patients diagnosed with PAP, who had hypoxia or worsening disease, were recruited between 2003 and 2011 from the Department of Respiratory Medicine. Patient history, physical examinations and other data were collected at the time of diagnosis, when possible.

Results from the bronchoalveolar lavage (BAL), characteristic high resolution-CT (HR-CT) and/or histopathological observations from biopsies were used as the basis for diagnosis. In the present study, transbronchial lung biopsy was used to diagnose 3 patients. PAP was also diagnosed using surgical lung biopsy (4 patients) and BAL (3 patients). Additionally, 1 patient was diagnosed using BAL at the Zhongshan Hospital of Fudan University (Shanghai, China).

### Disease severity score (DSS)

A PAP DSS was assigned to each patient. This score was based on the presence of symptoms, including dyspnea or cough, and the grade reduction of PaO_2_, as previously described (8,9).

### Pulmonary function tests and arterial blood gas analysis

Measurements were obtained from each patient, including the maximum vital capacity (VCmax), forced expiratory volume in 1 second (FEV1), forced vital capacity (FVC) and diffusing capacity for carbon monoxide (DLCO). Arterial blood gas was also analyzed. To measure the pulmonary function, the Jaeger MS-PFT and MS-Diffusion spirometer (Jaeger, Hoechberg, Germany) were used, and all the processes were handled by the same technician. The pulmonary function tests were performed when the patient was admitted to hospital and two weeks after WLL. The arterial blood gas analysis was conducted by the clinical laboratory with a GEM Premier 3000 blood gas analyzer (Beckman Coulter, Miami, FL, USA) prior to and following WLL. The blood was obtained from the radial artery at room temperature.

### WLL

In order to determine whether WLL is required, universal indicators were used, which include severe dyspnea and/or hypoxemia and a PaO_2_ level of <60 mmHg. The risks associated with WLL include those specifically associated with general anesthesia, double-lumen endotracheal intubation and mechanical ventilation necessary for the procedure. Additionally, there are risks associated with the lavage itself, as well as the requirement for continued ventilatory support post-procedure and monitoring in a critical care setting. Electrocardiogram as well as tests of coagulation, liver and kidney functions were performed prior to the procedure, which was then performed in the operating room.

**Figure 1:**
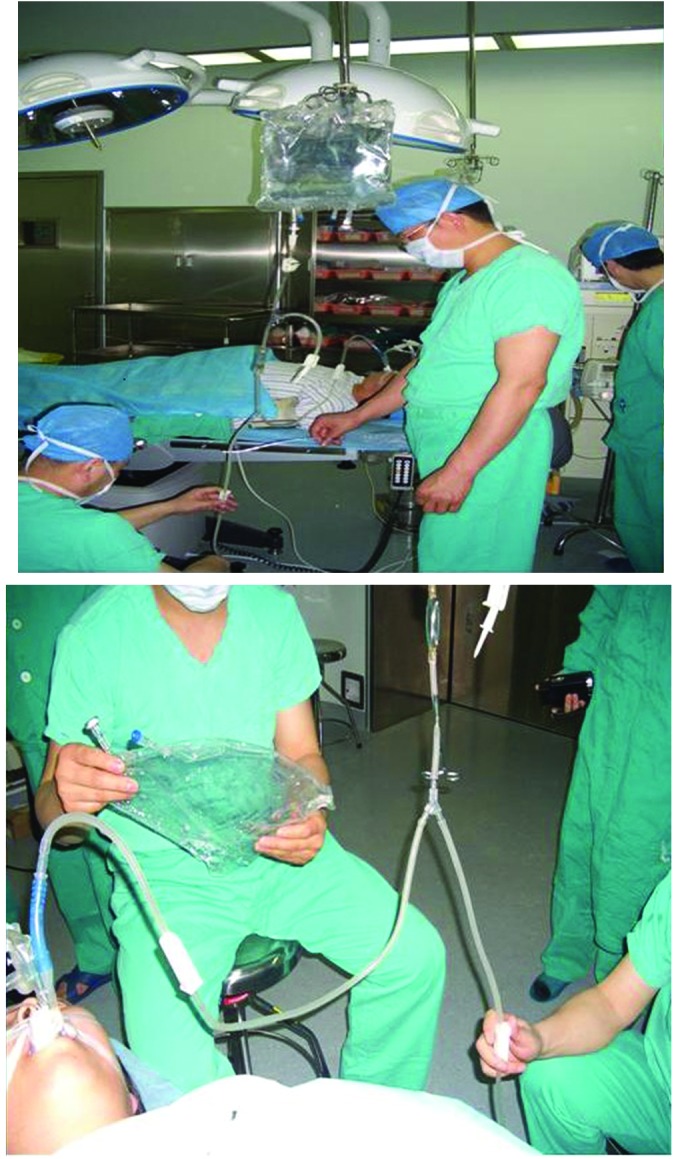
Whole lung lavage procedure.

Therapeutic alveolar lavage of either the right or left lung was performed under general anesthesia and paralysis. Cardiopulmonary monitoring was performed to ensure that the process was smooth and that no complications occurred. The patient was intubated with a double-lumen endobronchial tube and a flexible bronchoscopy was performed simultaneously to confirm the appropriate tube placement. The bronchial and tracheal balloons were inflated to isolate the lungs and intermittent positive pressure ventilation was initiated with a volume of 400 ml at a rate of 13 breaths/min. While WLL was performed in one lung, the other was ventilated. The WLL procedure is demonstrated in Fig. 1.

**Figure 2:**
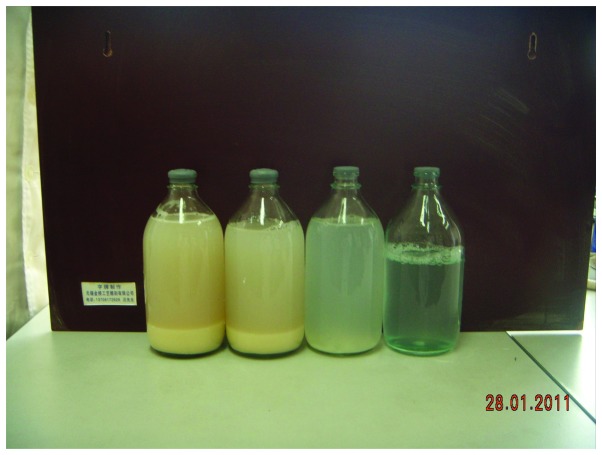
Lavage fluid from the whole lung lavage procedure.

The patients were placed on an operating table in the lateral decubitus position, with the lung being lavaged in the nondependent position (up) in order to reduce the perfusion of the treated lung (thus providing an improved ventilation/perfusion ratio). Sterile 0.9% NaCl warmed to body temperature was used as lavage fluid. Prior to the lavage procedure, ~200 ml saline was instilled to fill the lung up to the functional residual capacity. Subsequent to this, aliquots between 500 and 600 ml saline were instilled to the lung. Saline bags were placed ~60 cm above the table surface, allowing the fluid to flow into the lungs by gravity. Following each instillation, the fluid was drained passively by positioning the operation table, with the addition of concomitant chest percussion. The recovered fluid was collected via a 2-way stopcock into 500 ml bottles to allow turbidity to be assessed. The procedure was repeated until the effluent, which was initially opaque and milky, became clear (Fig. 2). A total of ~6,000–26,000 ml of warm saline was poured into the lung. The residual saline solution was then aspirated as far as possible.

Following pallanesthesia, the patient was extubated and transferred to the hospital ward. The lavage of the other lung was performed following an interval of 4–10 days.

### Ethical considerations

The present study was approved by the Ethics Review Committee of Wuxi People’s Hospital and Nanjing Medical University. Informed consent was obtained from all participants following a detailed description of the purpose and potential benefits of the study.

### Statistical analysis

All statistical analyses were performed using SPSS statistical software version 19.0 (SPSS, Inc., Chicago, IL, USA). The paired t-test was used to compare the differences between continuous variables prior to and following WLL, whilst the Wilcoxon T test was employed to compare the categorical variables. P<0.05 was considered to indicate a statistically significant difference.

## Results

### Demographics

The age at diagnosis ranged between 31 and 66 years, with a mean age of 48.3 years. A male predominance (eight males versus three females) was observed in the present study. Detailed demographics of the study population are illustrated in Table I.

### Diagnostic methods

PAP was diagnosed using either surgical lung biopsy (4 patients) or BAL (3 patients) at the Wuxi People’s Hospital Affiliated to Nanjing Medical University. One patient was diagnosed using BAL at the Zhongshan Hospital of Fudan University. The remaining patients were assessed using HR-CT scans to observe characteristic ‘crazy-paving’.

### Smoking habits

In total, 8 out of 11 patients (72.7%) were current or ex-smokers (75% current, 25% previous; Table I). All the females had never smoked before. All active smokers at the time of diagnosis reported a heavy consumption of >20 cigarettes/day.

### Symptoms and DSS

Among the symptoms reported at the time of diagnosis, the majority of PAP patients complained about dyspnea (90%), followed by cough (27.3%) and sputum (27.3%). Physical examination revealed acropachy in one case. The distribution of the patients according to DSS grades is shown in Table I. The majority of patients had DSS scores of 4 and 5.

### Analysis of disease course and WLL

The follow-up time ranged between two and eight years. Following WLL, all patients showed an improvement in respiratory symptoms and the survival rate was 100%. The majority of patients received WLL once, however, one patient underwent WLL three times between 2003 and 2009 and another patient underwent WLL twice within two years. In all patients, no severe adverse effects occurred during and subsequent to WLL.

**Figure 3:**
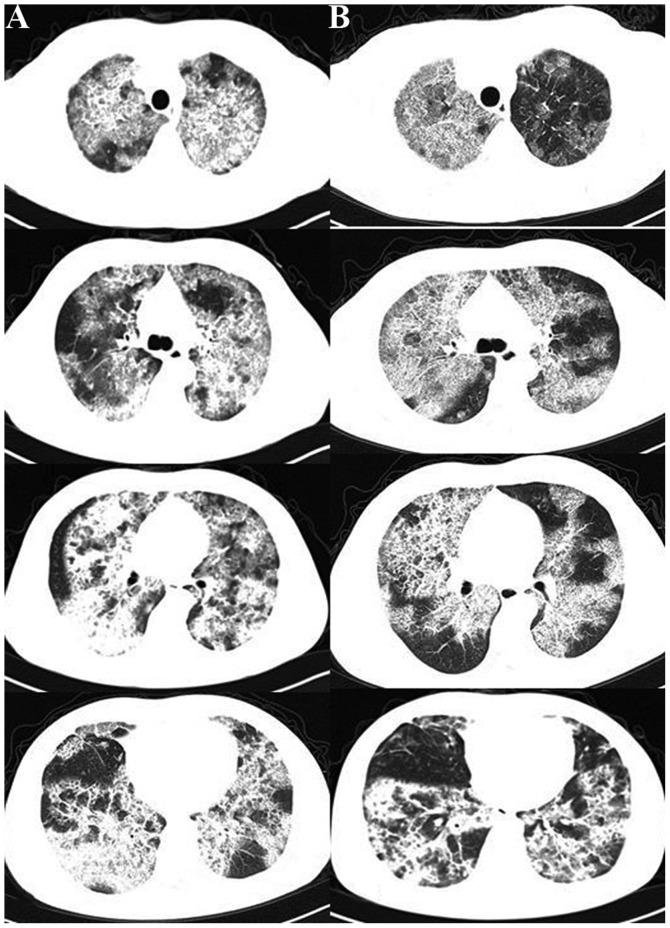
Computed tomography scan image of pulmonary alveolar proteinosis (A) prior to and (B) following the whole lung lavage.

The levels of oxygen markedly improved following therapeutic lavage. The mean PaO_2_ was 54.8±7.4 mmHg prior to WLL, which increased to 68.0±8.5 mmHg following WLL. The PaO_2_ following WLL showed moderate improvement (P=0.010), while the D(A-a)O_2_ also improved following WLL (P=0.028; Table II). Pulmonary function tests were performed two weeks subsequent to WLL for nine patients. All but one patient had a slight improvement in DLCO (P=0.000; Table III). VCmax improved in four cases and worsened in five cases. No significant difference in the mean VCmax (% predicted) prior to and following WLL was identified. The mean FVC (% predicted) and FEV1 (% predicted) decreased following WLL, and no significant differences were detected in FVC (% predicted) and FEV1 (% predicted) prior to and following WLL (P=0.072; Table III). Chest radiographs revealed that all but one patient showed a significant improvement following WLL. A representative CT scan image is shown in Fig. 3.

## Discussion

In the present study, the characteristics of a total of 11 patients with PAP were described. The median age at diagnosis in the present study was 48 years, which is higher than the mean age observed in cohorts analyzed by Seymour *et al* (10), Xu *et al* (11), Bonella *et al* (12) and Campo *et al* (13), but lower than the cohort investigated by Inoue *et al* (9). The male to female ratio was 2:7, which was similar to the ratio observed by Seymour *et al* (10). Furthermore, the present study revealed that the majority of patients with PAP were smokers (current or former), however, a significant portion of patients with PAP had never smoked in other cohorts, ranging between 21 and 43% (9,12).

PAP is a rare diffuse pulmonary disease characterized by the accumulation of PAS-positive lipoproteinaceous material, predominantly phospholipid surfactants and surfactant apoproteins, in the distal air spaces, resulting in impaired gas transfer. PAP is frequently mistaken for interstitial lung disease. The presence of macroscopic milky fluid and/or the presence of amorphous, eosinophilic, PAS positive material, the presence of lipid laden macrophages on BAL analysis as well as the appearance of a ‘crazy paving pattern’ in the high resolution CT scan images of the thorax, are all used as diagnostic tools. In the present study, the diagnosis of PAP was confirmed by either surgical lung biopsy (4 patients) or BAL (4 patients). Three patients were assessed by typical ‘crazy paving’ in a HR-CT scan.

Among the symptoms presented at the time of diagnosis, the majority of patients with PAP complained about cough, sputum and dyspnea, which is consistent with the results observed in Germany and Japan (9,12). The DSS is a useful tool to assess disease severity, as previously described by Inoue *et al* (8,9) and Bonella *et al* (12). In the present study, the majority of patients had a DSS of 4 and 5, which indicated a worsened disease severity of PAP patients.

Given the recent insights into the pathogenesis of the disorder and the role of GM-CSF in the pathophysiology of PAP (14), novel targeted biological therapies have been proposed and have been reported in previous studies (15,16). In China, investigation into GM-CSF therapy is ongoing.

The success rate for GM-CSF is not yet sufficient to replace WLL therapy, despite increasing evidence suggesting that GM-CSF therapy may be beneficial for patients with PAP (12). As mentioned above, WLL to date represents the conventional therapeutic treatment for PAP (17–20).

There are no clearly established criteria for when to perform WLL; all patients with PAP who had hypoxia or worsening disease were recruited, and the majority of patients had DSSs of 4 and 5 in the present study.

In the present study, the WLL technique was performed at Wuxi People’s Hospital Affiliated to Nanjing Medical University under general anesthesia in an operating room. The patient was intubated with a double lumen endotracheal tube and fiber-optic bronchoscopy was performed to confirm the appropriate tube placement. The patient was placed in the lateral decubitus position and the lung was lavaged in the uppermost position, while the non-lavaged lung was mechanically ventilated. The injection of warmed (37°C) saline into the lung was performed and, following opening the outflow tube, the fluid was collected. In order to improve drainage, manual chest percussion, which significantly augments the removal of proteinaceous material, may be performed. Chest wall percussion was conducted once the outflow, which was initially milky, became clear. Lavage and percussion continued until the outflow fluid became definitively clear.

The efficacy of WLL in the present study was evaluated by analyzing the patient symptoms, DSS, arterial blood gas, pulmonary function tests and radiological findings. All patients showed symptomatic, radiographic and functional improvement following WLL. Subsequent to the therapeutic lavage, oxygenation parameters, including PaO_2_ and D(A-a)O_2_, markedly improved in comparison with the initial examinations, which is consistent with results observed in previous studies by Byun *et al* (21) and Bonella *et al* (12). The DSS distribution of the patients also improved, with the majority of the patients having a DSS of 3. Although the data from the present study revealed that the diffusing capacity of the lung was improved but not the ventilation, due to the small sample size, further studies are required with a larger sample size over a longer time period to confirm the present results. WLL can reduce the area of high-density reflection on CT scans. All patients with PAP who underwent WLL survived to the end of the present study. In addition, in a long-term follow up of the 11 patients with PAP (collected between 2003 and 2011), it was found that WLL was safe and efficacious, providing long lasting benefits.

In conclusion, WLL improves the survival rate and is an effective approach for the treatment of PAP, which may significantly improve clinical symptoms, blood gas analysis and radiographic features.

## Figures and Tables

**Table I:** Demographics and disease features at diagnosis.

Characteristic	Value
Gender, n (%)	
Male	8 (72.7)
Female	3 (27.3)
Age, years (mean ± SD)	48.3±10.8
PAP type, n (%)	
Primary	11 (100.0)
Secondary	0 (0.0)
Smoking habits, n (%)	
Never	3 (27.3)
Previous	2 (18.2)
Current	6 (54.5)
Radiographic PAP features, n (%)	
Characteristic	11 (100.0)
Non characteristic	0 (0.0)
DSS grade, n (%)	
1	0 (0.0)
2	1 (9.1)
3	1 (9.1)
4	5 (45.5)
5	4 (36.4)

SD, standard deviation; PAP, pulmonary alveolar proteinosis; DSS, disease severity score.

**Table II:** Pre- and post-lavage arterial blood gas analysis (mmHg).

Patient number	PaO_2_ (mmHg)		D(A-a) O_2_ (mmHg)	
	
	Pre-lavage	Post-lavage	Pre-lavage	Post-lavage
1				
Lavage 1	54.8	78.0	53.1	12.3
Lavage 2	63.2	60.2	31.8	39.2
Lavage 3	53.0	60.0 (9 l/min)	66.0	312.0
2	55.3	83.1	57.8	24.2
3	52.0	-	29.0	-
4	34.0 (2 l/min)	47.0	133.0	71.0
5				
Lavage 1	56.0 (2 l/min)	-	120.0	-
Lavage 2	49.0	69.0	62.0	42.0
6	58.1	63.0	21.8	15.7
7	49.5	57.0	28.9	17.4
8	57.0	72.4	34.0	21.6
9	52.0	56.0 (2 l/min)	52.0	124.0
10	41.0	67.0	30.0	16.8
11	65.0	62.7	45.0	48.5

**Table III:** Pulmonary function tests pre- and post-lavage

	FEV1 (% Pred)		FVC (% Pred)		VCmax (% Pred)		DLCO (% Pred)	
				
Patient	Pre-lavage	Post-lavage	Pre-lavage	Post-lavage	Pre-lavage	Post-lavage	Pre-lavage	Post-lavage
1								
Lavage 1	86.4	88.2	82.1	83.1	78.8	80.2	49.4	60.6
Lavage 2	93.5	93.2	79.3	78.6	76.1	76.2	-	57.8
Lavage 3	85.6	86.4	78.2	80.5	76.0	77.9	-	61.4
2	98.6	97.6	90.0	89.7	86.9	83.7	49.5	54.2
3	104.6	91.7	74.1	75.2	99.4	87.9	64.2	67.9
4	-	-	-	-	-	-	-	-
5								
Lavage 1	-	57.4	-	54.8	-	60.7	-	34.2
Lavage 2	69.0	70.2	61.3	60.7	62.0	61.9	30.8	32.4
6	101.0	-	96.2	-	109.5	-	69.6	-
7	46.0	43.8	40.3	43.8	38.6	40.3	23.8	27.4
8	88.1	76.6	73.7	69.2	72.1	67.3	55.5	59.1
9	106.3	94.6	92.6	87.5	22.9	31.7	62.4	59.5
10	69.8	67.3	68.6	66.4	69.3	57.4	46.6	56.3
11	68.0	68.4	69.9	70.2	67.0	67.2	29.5	32.4

% Pred, percentage of the predicted value; FEV1, forced expiratory volume in 1 second; FVC, forced vital capacity; VCmax, maximum vital capacity; DLCO, diffusing capacity for carbon monoxide.
